# Surgical management of glaucoma: An Indian perspective

**DOI:** 10.4103/0301-4738.73697

**Published:** 2011-01

**Authors:** R Ramakrishnan, Mona Khurana

**Affiliations:** Glaucoma Services, Aravind Eye Hospital and Postgraduate Institute of Ophthalmology, Tirunelveli, Tamil Nadu - 627 001, India

**Keywords:** Glaucoma, glaucoma drainage devices, Indian scenario, shunts, trabeculectomy

## Abstract

Glaucoma is a serious sight-threatening disorder aptly named the *Silent thief of sight*. India, being the second most populous country in the world, has about 20% of the world glaucoma population. The complex geographical and socioeconomic architecture and the economic extremes have a profound effect on its health system. The present times are abundant with fresh developments in the field of glaucoma. Though newer modalities are present in India, they are not ample and are unequally distributed. Adherence and persistence with medical therapy is an issue owing to a multitude of factors. In such a setting, most of the ophthalmologists find themselves performing glaucoma surgeries quite often. In the present era, there are a number of new surgeries to choose from, especially procedures which are nonpenetrating and blebless. Faced with a spectrum of surgeries from shunts to canal surgeries and trabecular bypass devices, the surgeon is often in a dilemma. Still, trabeculectomy remains the gold standard with an increasing trend toward glaucoma drainage devices. The new procedures and devices are worth exploring but await long-term results, good training of surgeons and cost effectiveness.

Glaucoma is a serious sight-threatening disorder aptly named the *Silent thief of sight*. It is an optic neuropathy with progressive loss of retinal ganglion cells, leading to characteristic structural damage to the optic nerve and visual field defects due to a variety of pathologies.[1] It is the second leading cause of blindness in India and the country has been predicted to host nearly 20% of the world glaucoma population by 2020.[2,3] It was estimated that 12 million Indians will be affected by 2010. Now, at the dawn of a new decade, we are looking at a challenging estimate of 16 million by 2020.[2]

Various well structured studies give an estimate of the magnitude of the problem in India. The reported prevalence of Primary Open Angle Glaucoma (POAG) is 0.41–3.51%.[4–10] Population-based studies have reported Primary Angle Closure Glaucoma (PACG) to be almost as common as POAG in India.[4–10]

India is the second most populous country in the world with an increasing aging population. It is diverse, multicultural and undergoing rapid but unequal economic growth. With its complex social architecture and economic extremes, the effect on health system is multifold. There has been a definite growth in the overall healthcare resources and health-related manpower in the last decade in India. At the same time, pre-existing inequality in the healthcare provisions has also increased. The socially underprivileged are unable to access healthcare due to geographical, social, economic or gender related factors.[11]

The treatment of glaucoma is currently directed toward lowering of intraocular pressure (IOP) to prevent or slow progression of optic nerve head damage (evidence from randomized control trials).[12–15] The modalities to regulate IOP are medical, laser-assisted therapy and filtering surgeries. The present article discusses the surgical management of glaucoma from an Indian perspective.

## Are We Ready to Meet the Challenge?

Many of the newer diagnostic modalities for early diagnosis and monitoring progression of glaucoma are available in the country. The spectrum of antiglaucoma medications is readily available. Indian manufacturers have made a number of drugs available at affordable prices. Several public and private hospitals are equipped to provide state-of-the-art care to the patients and high quality training to residents. Yet, more than 90% of the glaucoma remains undiagnosed contrary to 40–60% in developed countries.[4–10,16] Less than one fifth of those with glaucoma in the Aravind Comprehensive Eye Survey (ACES) had been previously diagnosed as having the disease despite an eye examination in the past.[6] In Chennai Glaucoma Study, a significant number (40%) diagnosed as POAG actually had PACG.[4]

Healthcare resources in India, though ample, are inadequate. There were just 12,000 ophthalmologists, i.e., 1 per 100,000, with very few glaucoma specialists in 2001.[16,17] Most ophthalmologists in India (70%) are located in urban areas and cater to only 23% of its population.[15,18] Many ophthalmologists do not practice comprehensive eye care as the quality of residency training is extremely variable with very few institutes offering structured glaucoma fellowships.[19,20]

## The Patient’s Perspective

A large percentage of blindness in our country stems from the population living in the rural areas where medical facilities are not easily available. To compound this further, glaucoma is an asymptomatic disease with no appreciable benefit to the patient with therapy. The side effects of medicines may lead to a decreased quality of life. The need for lifelong treatment in spite of lack of improvement in their vision does not motivate most patients. Thus, adherence is an issue. Nearly 35% of the Indian population falls below the international poverty line. So, in spite of the availability of medications at lower prices than the West, they are still not affordable considering the lifelong need. In ACES, 42% of glaucoma patients reported one or more problems in using the medications.[6] This makes glaucoma a cost-intensive disease, with a low socioeconomic status having a negative impact. The added expense of prolonged, regular antiglaucoma medications is a major hurdle.

## Surgery: The “Cutting” Edge

Considering the above points, many ophthalmologists find themselves performing glaucoma surgery quite often, dictated by issues like socioeconomic status and adherence. Moreover, the benefit of a more optimal lowering of IOP and a better diurnal control is provided by surgery.[14,21]

An ideal surgery is the one which can be easily performed by all surgeons, requiring simple instrumentation with minimal complications, and is replicable with a short learning curve. Moreover, it should be economical and provide long-term success. On the road to achieving this ideal, any new surgical procedure should have some added benefit over the pre-existing one.

## Trabeculectomy

**Figure 1:**
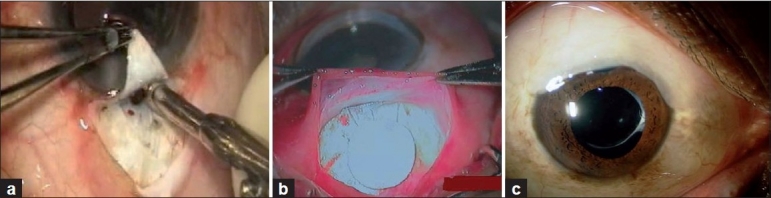
(a) Trabeculectomy ostium being fashioned with a Kelly Descemet punch. A fornix based conjunctival flap has been made.(b) Trabeculectomy with a collagen implant. (c) A diffuse well functioning bleb after a combined single site phacotrabeculectomy with mitomycin C

Sugar’s trabeculectomy (1961), popularized by Cairns, was quickly adopted after it rivaled the success of full-thickness procedures, with fewer complications and an effective lowering of IOP.[22] Further variations in technique, the introduction of antimetabolites, collagen implants, releasable sutures, laser suture lysis and anti VEGFs (anti vascular endothelial growth factor) yielded even better results [Fig. 1a and b]. As a result of the above, the success rate of modern trabeculectomy in experienced hands is estimated between 60 and 100%, depending on patient selection, definition of success and length of follow-up.[23] But then, as early postoperative complications related to wound leak, hypotony, and late-onset complications associated with the bleb, antimetabolite use and failure began to emerge, surgeons started looking for alternatives.

The lack of consensus regarding the best procedure for different groups of patients was underscored by two surveys. The first, a 1996 survey of both the American and Japanese glaucoma societies, concluded that the majority of respondents preferred trabeculectomy with mitomycin C (MMC).[24] Six years later, trabeculectomy was still the preferred treatment in most scenarios, but use of glaucoma drainage implants had increased significantly.[25]

Surgeons in India often perform early or even primary trabeculectomy owing to logistics of adherence to therapy, socio economic status or patients presenting at an advanced stage when target IOP cannot be achieved with medications. In a poll of glaucoma specialists and general ophthalmologists conducted at the Glaucoma Society of India meeting (November 2000), a majority favored trabeculectomy with the concurrent use of MMC.[19] When combining trabeculectomy with cataract extraction, phacotrabeculectomy (single or two site) is the preferred technique [Fig. 1c]. Trabeculectomy is also frequently combined with manual small incision surgery and extracapsular surgery.[19] The indication for a combined surgery on an average is earlier in the lower socioeconomic groups due to adherence factors and lifelong expense of therapy.

## Glaucoma Drainage Devices

Improved designs and data from new trials make glaucoma drainage devices (GDDs) an interesting option. Over the years, advances in shunt technology, especially in the use of more biocompatible materials, have led to improvements in the original Molteno design along with the development of Baerveldt shunt and the popular Ahmed Glaucoma Valve (AGV). These devices historically had been reserved for refractory glaucomas at high risk of failure with standard filtering surgery, mainly as a surgery of last resort. Results from the Tube Versus Trabeculectomy (TVT) study have defused the current bias against drainage implants in eyes with previous cataract surgery. The 3-year results of TVT study provide further evidence that the role of tube shunts in the surgical management of glaucoma should be expanded.[26] Glaucoma Drainage Devices have attained success rates ranging between 25 and 94%, most commonly above 60%, depending on the type of shunt used, the definition of success criteria, the length of follow-up and the characteristics of the population studied.[27] While there is still no consensus about which of the two commonly performed glaucoma operations is better for patients, most glaucoma specialists agree on the pros and cons of each technique.

### Pros of trabeculectomy

Trabeculectomy is a highly successful time-tested surgery. In good hands, it typically achieves low IOP from day 1, without medication. Relatively predictable and a straightforward technique, it is also cost effective.

### Cons of trabeculectomy

- Bleb-related complications: Bleb leaks and infections (blebitis and endophthalmitis)
- Hypotony and hypotonous maculopathy (with antimetabolite use)
- Poor prognosis in the presence of conjunctival scarring

### Pros of GDD

- Low risk of late infection
- IOP-lowering effect may be longer lasting
- Straightforward technique
- May be implanted in eye with scarred conjunctiva
- Less bleb-related complications
- Allows use of contact lenses postoperatively

### Cons of GDD

**Figure 2:**
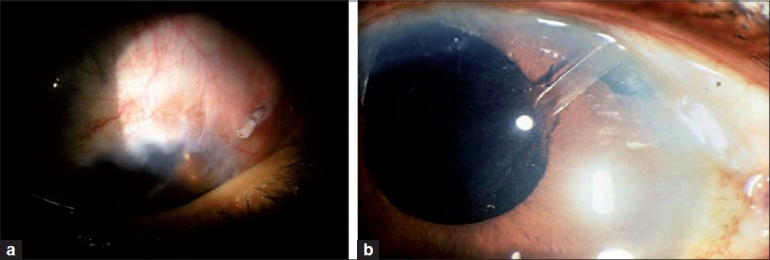
The glaucoma drainage devices come with a new set complications (a) Tube retraction and exposure (b) Vitreous Blocking the tube of an AGV

- Cost
- A set of unique tube-related complications like corneal decompensation (in case of tube corneal touch), conjunctival erosion, tube retraction [Fig. 2a], implant exposure and tube obstruction [Fig. 2b]
- Hypertensive phase
- May not lower pressure as well as trabeculectomy
- May require supplemental medical therapy to achieve the desired pressure
- Cosmetic concerns regarding the “bleb” over the shunt and the patch graft being visible
- Occular motility disorders and diplopia: When permanent can be very disabling and may require prism glasses or surgery
- Hypotony in case of nonvalved devices
- Encapulation and late failure

The shunts vs. trabeculectomy comparison actually began in the late 1960s and early 1970s. Almost 40 years later, trabeculectomy still remains the “gold standard” surgical option for glaucoma, with GDDs being used in complicated cases where trabeculectomy has failed or where trabeculectomy is not a viable option. This second group comprises patients with neovascular glaucoma, severe inflammatory glaucoma and those with scarring of the conjunctiva where a standard trabeculectomy would not work. Presently, this spectrum is expanding.

Today, the most widely used GDDs are the Ahmed valve and the Baerveldt glaucoma implant. There is general agreement that there is no single “best” glaucoma drainage device, with the choice determined by the surgeon’s preference and the patient’s individual needs. An Ahmed glaucoma valve has a valve that is designed to prevent hypotony in the immediate postoperative period. It has a significant hypertensive phase, peaking at 1–2 months and resolving by 6 months. Studies in the Indian population have shown an effective and sustained control of IOP with AGV.[28,29]

The Baerveldt requires a careful surgical technique and frequent follow-up. However, it attains lower IOP than the Ahmed valve owing to its larger surface area. Choroidal effusions are among the most common complications seen.[30] Attempts at manufacturing more economical GDDs are ongoing in the country.

## New Trends: “Blebless” Surgery

The ideal is a blebless surgery, which lasts long and keeps the pressure down. There are quite a few promising procedures with encouraging results. The concept of these procedures is to make the Schlemm’s canal more accessible to aqueous or bypass it.

Nonpenetrating filtering procedures reduce IOP by enhancing the aqueous outflow natural aqueous outflow channels, while reducing outflow resistance located in the inner wall of the Schlemm’s canal and the juxtacanalicular trabecular meshwork. They facilitate the aqueous egress through an intact Descemet’s membrane. These include *Deep Sclerectomy and Viscocanalostomy*. Both procedures unroof the Schlemm’s canal and rely on the flow of aqueous through a thin trabeculo-Descemet’s window, theoretically eliminating the dependence on conjunctival healing.[31,32] Deep Sclerectomy can be done with or without collagen implants. Subsequent goniopuncture may be required. However, most versions of deep sclerectomy rely on the presence of an intrascleral filtering bleb. They are technically difficult to perform, with a risk of late scarring and are not without complications. Recently, to overcome the long learning curve of deep sclerectomy, a variety of lasers including Carbon dioxide laser are being tried to ablate the deep scleral tissue.

**Figure 3:**
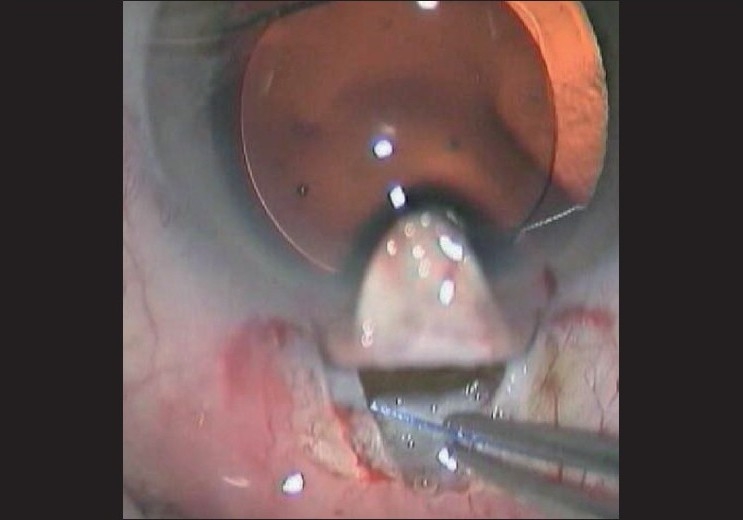
Canaloplasty: A nonpenetrating, blebless procedure involving dilation and suture tensioning of the entire Schlemm’s canal (Image: Courtesy Dr. Gábor Scharioth)

The *Glaukos iStent* a titanium device placed inside the Schlemm’s canal, allows the aqueous humor to flow directly into the canal, bypassing the trabecular meshwork.[33] It is inserted via a clear corneal incision under topical anesthesia and has the advantage of being devoid of a bleb and associated complications. The *Gold Microshunt* (GMS), a biocompatible gold shunt implanted in the suprachoroidal space, uses the eye’s natural pressure differential (uveoscleral outflow) to divert the aqueous into the suprachoroidal space in a controlled fashion. It has the advantage of postoperative phototitration with a laser.[34] The *Ex-PRESS glaucoma filtration device*, a small stainless steel device, is now most often implanted under a large partial thickness scleral flap. It lowers IOP effectively but has bleb-related complications.[35] *Canaloplasty*, a variation of viscocanalostomy involves circumferential catheterization and viscodilatation of the entire length of the Schlemm’s canal, thus restoring the natural trabeculocanalicular outflow passage and effective lowering of the IOP in POAG.[36] An adjunct to the procedure involves placing a prolene suture in the canal to keep it open [Fig. 3]. The *Trabectome* uses a microelectrocautery to ablate a strip of trabecular meshwork and the inner wall of Schlemm’s canal with a focused electrosurgical pulse. This provides direct access of aqueous to outflow channels. Done mostly in POAG, it provides a reasonable IOP reduction, a significant decrease in medications and can be combined with phacoemulsification.[37,38]

Lasers are also becoming increasingly popular with *Excimer laser trabeculostomy* being used to create small holes into inner wall of Schlemm’s canal via the anterior trabecular meshwork, with minimal thermal effects and lack of coagulative damage. *Endocyclophotocoagulation* (ECP) involves photocoagulation of the ciliary processes under direct visualization and is usually combined with cataract surgery. Encouraging results have been reported in a study on Indian subjects with refractory glaucoma.[39]

Advantages that most of these procedures offer are a clear corneal approach with preservation of conjunctiva for future glaucoma surgery, less incidence of early postoperative complications and the absence of a bleb and related complications. Some are non penetrating or minimally penetrating with no conjunctival bleb and no need of an iridectomy.

The entire concept is attractive and appears promising. However, one must keep in mind certain limitations of these procedures such as the additional cost, a long learning curve and use in a limited spectrum of glaucoma. Inadequate training plus a variety of new techniques further add to the confusion. The safety profile is superior but the amount of pressure lowering is moderate. In terms of efficacy, they cannot compete with excellent IOP reduction achieved with a trabeculectomy. Moreover, the Schlemm’s canal may not be entirely healthy and lacks a circumferential flow. All these procedures await long-term results, randomized control trials and cost effectiveness.

## Conclusion

The evidence to date still suggests that there is a greater likelihood of lower IOPs being achieved by *“penetrating”* surgery.[40] It is quite common to require one or two anti glaucoma medications to control the IOP long term after a glaucoma drainage device. It is also quite common to have a “hypertensive phase” in the postoperative period in case of AGV or hypotony in case of Baerveldt. The added set of new complications adds to the cost and decreased quality of life. Long-term results are still awaited.

As of now trabeculectomy appears as the better option for the masses in the Indian scenario yet, it is not the final solution. New surgeries offer hope and require careful patient selection and counseling. A thorough understanding of the risks and benefits is also essential. No matter what treatment option one offers to the glaucoma patient, constant follow-up and monitoring has to be emphasized.

A surgery which is technically simple and promises to be long lasting and is much less expensive is of value to economically developing nations where the technical requirements of man and machine are not very easy to obtain all over.

Osler, a great physician, said *“don’t be the first to try something and don’t be the last to give something up”*. The ideal scenario is that the surgeon should be honest with the patient and perform the surgery the surgeon is best at doing.
